# Thermomechanical Processing of Medium-Carbon Boron-Bearing Microalloyed-Steel Forgings Targeting Normalized-like Structure and Properties

**DOI:** 10.3390/ma18214871

**Published:** 2025-10-24

**Authors:** Piotr Skubisz, Piotr Micek, Stanisław Flaga

**Affiliations:** 1 Faculty of Metals Engineering and Industrial Computer Science, AGH University of Krakow, al. A. Mickiewicza 30, 30-059 Cracow, Poland; 2 Faculty of Mechanical Engineering and Robotics, AGH University of Krakow, al. A. Mickiewicza 30, 30-059 Cracow, Poland; micek_pt@agh.edu.pl (P.M.); stanislaw.flaga@agh.edu.pl (S.F.)

**Keywords:** direct cooling, microalloyed steel, boron-bearing steel, 35MnTiB4, thermomechanical processing, hot forging, normalizing annealing

## Abstract

The paper presents designing thermomechanical processing routes for medium-carbon boron-bearing microalloyed steel and investigates their effect on microstructure–property characteristics obtained through controlled cooling directly from hot forging temperature. Direct cooling was carried out in situ within the industrial process of hot forging, replacing conventional heat treatment with slow and accelerated air cooling, realized with a fully automated fan-cooling laboratory conveyor which accommodates the desired cooling strategy. Comparative analysis of conventionally normalized and direct-cooled microstructure and mechanical properties obtained under varied thermo-mechanical conditions is presented to investigate the potential of medium-carbon microalloyed steel with boron addition for producing tailored properties comparable to those of the normalized condition. The obtained microstructure composed of grain-boundary ferrite and pearlite which resulted in tensile properties as good as Re ≈ 610 MPa, Rm ≈ 910 MPa, and elongation A5 ≥ 12%. Although the achieved microstructure–property parameters differ from those achieved through conventional normalizing (Rm ≤ 780 MPa, Re ≤ 460 MPa, and A ≥ 14%), they are considerable in terms of selected machinability aspects. The observed effect of the imposed treatment strategies on interlamellar spacing and morphology of ferrite showed possibilities regarding the control of mechanical properties and application of direct cooling as a beneficial alternative to conventional normalizing, where energy consumption is the main concern in manufacturing high-duty parts made of boron-bearing microalloyed steel 35MnTiB4.

## 1. Introduction

Meeting ever-increasing performance demands and cost balance requirements in the mass production of high-duty components relies on die forging technology, which is indispensable in combining high strength and plasticity. Die forging has ceased to be merely a process aimed at obtaining a desired shape with greater or lesser accuracy. Competition between markets and producers gave rise to competition between manufacturing techniques, and it has been decades now that the forging process is aimed at both shaping and tailoring the final operational properties. Thus, parallel to the newest achievements in material science, conventional treatments are still modified to couple societal needs with production cost and benefits [1]. As opportunities for reducing production costs diminish and energy expenses continue to rise, thermomechanical controlled processing (TMCP) combined with alloy modification has emerged as an effective approach to cost reduction, providing both material and economic advantages [2]. In the background of pursuing increased strength properties to accommodate higher loads, efforts have been made to utilize conventional techniques for energy savings, reduction in emissions, and media consumption in the mass production of automotive and civil engineering products [3]. Based on the knowledge of the fundamentals of physical phenomena in alloys and contemporary technologies for predicting and controlling transformations in steel [4], TMCP is also successfully implemented in forging processes.

Utilization of the heat attained in the workpiece after forging allows avoiding multiple reheating of the as-forged part. In addition to reducing production costs, it enables control of the cooling process for fully or partially recrystallized structures, without adverse effects on final mechanical properties due to grain coarsening or precipitate coagulation. Applying direct cooling to microalloyed steel enables the replacement of traditional quenching–tempering treatments, producing a wide range of microstructures and superior mechanical properties that are characterized by a combination of high strength and ductility, with significant energy savings and efficiency. Its effectiveness and final properties depend strictly on determining the proper cooling rate and controlling it. While TMCP is successfully implemented in forging technologies to realize a variety of treatments, its potential to replace conventional treatments oriented at the softening of steel is not practiced, although theoretical basis and technical knowledge enable continuous normalizing of massive parts under controlled cooling. The cooling rate significantly influences the amount of pearlite and the size and spacing of the pearlite lamellae [5,6]. At higher cooling rates, more pearlite forms, and the lamellae are finer and more closely spaced. The increased amount of pearlite and the greater fineness of the pearlite result in higher strength and higher hardness. Conversely, lower cooling rates result in better impact strength [7]. In any part with thick and thin sections, the potential exists for variations in cooling rate, and thus for variations in strength and hardness [8].

Die forgings are ordered in the as-forged, normalized, or heat-treated condition. If forgings are ordered as semi-finished products, the manufacturing chain is designed with the assumption that further machining will follow [9]. Achieving the final shape of a product through forging is very difficult. To a greater or lesser extent, regardless of forging accuracy, die forgings contain a few allowances—the excess material that needs to be removed by machining [10,11]. The allowances resulting from: draft angles, compensation for forging inaccuracies, edge rounding, mismatch, the need to fill cavities or undercuts to allow removal of the forging from the die, and subsequent machining due to the difficulty of achieving a good surface quality after hot forging, constitute surplus material that must be removed [12]. Therefore, machining necessitates a hardness level appropriate for cutting tool durability—this hardness depends on the microstructure’s structural composition and morphology [13]. Due to the limited controllability of recrystallization and grain growth processes in traditional hot forging [14], producing a microstructure that promotes good machinability after forging requires additional heating and cooling steps, such as normalizing, recrystallization, or spheroidizing annealing [15,16].

Normalizing is a heat treatment process intended to homogenize austenite after deformation or transformation of supercooled austenite, promoting a fine-grained microstructure throughout the material [17,18]. The definition of normalizing alone indicates that it must involve time and temperature. Thus, the same microstructural effect cannot be achieved by direct cooling from forging temperature. Microstructure uniformity is therefore a condition inherently tied to the traditional technology of normalizing. In principle, attaining its uniformity through controlled cooling is challenging, but it does not mean that direct cooling cannot substitute for it, providing comparable or considerable properties, especially hardness and machinability defined by other aspects than mere tool wear [19]. It is a fact that the acceptance standards for normalized forgings require fine grain size and microstructural uniformity. In fact, such a normalized structure provides only one aspect of machinability. In contrast, making the material soft does not exploit all essential considerations of good machinability, such as breaking the chip- or adhesion-related problems. Thus, plasticity improvement and other goals can be achieved through different processing routes, which may bring additional benefits, in addition to material condition. In a sense, the commonly used standards are somewhat held hostage to the traditional approach. Assuming traditional annealing is used to prepare the material for further processing, microstructural uniformity indicates that the annealing process was performed correctly. It is not easy to imagine not meeting this requirement by conventional normalizing. However, if the hardness requirements in the areas subject to machining are met, we can consider strengthening the regions not subjected to machining. Thus, from the utilitarian point of view, the key issue is to achieve a material state conducive to good machinability.

Provided that machining concerns the bulky sections, strengthening the material in areas not subject to machining can be advantageous. Most importantly, regardless of the expected or acceptable microstructural uniformity, replacing traditional normalizing with direct cooling, which provides equivalent mechanical properties, is economically beneficial, significantly reducing the energy costs associated with re-austenitization. In addition to financial benefits, it offers a wide range of possibilities for controlling the thermo-mechanical processing conditions to obtain the required mechanical properties in areas not subjected to machining. If hot deformation conditions and controlled cooling are properly designed and maintained in the process, the material–property requirements can be met without quenching–tempering heat treatment.

The example presented in this study illustrates an attempt at industrial implementation of controlled processing applied to a die forging made using a hammer, which is contrary to preceding studies that focus on utilizing the heat generated in a hot-forged part to provide controlled-rate direct cooling. Thus, tailored properties in hammer-forged parts [19,20,21,22], which are properties equivalent to those obtained through normalizing, are targeted. Contrary to appearances, producing a normalized-like structure through direct cooling is not an easy task. Different strengthening mechanisms call for proper selection and controlled implementation of time–temperature regimes during heating up, deformation, and subsequent cooling.

Contrary to treatments oriented at high strength, in this case, moderate hardness and good plasticity are of primary importance. This study aims to achieve the properties through controlled cooling in a continuous process. This involves the necessity of utilizing FEM analysis for selecting thermo-mechanical parameters that enable the realization of in situ experimental tests, considering the limitations of the industrial process.

## 2. Materials and Methods

### 2.1. Assumptions and Plan of the Study

The study aimed to investigate the effect of controlled cooling of hot-forged medium-carbon boron-bearing steel 35CMnTiB4 microalloyed with Ti, V, and Nb. The chemical composition of the investigated steel is designed to provide high strength to bulky parts. Due to its good hardenability, it is readily used where uniform properties are required in thick sections. To enable machining, it is normalized and subjected to heat treatment by quenching and tempering. The idea of the study was to achieve the targeted mechanical properties and microstructure by replacing reheating and subsequent air cooling with direct cooling immediately after hot forging. This approach is motivated by the fact that this steel is not an easy one to normalize, despite the conventional time-consuming process and the high energy consumption it causes. Thus, if a comparable effect were to be achieved without reheating, achieving the desired properties—equivalent or similar to those obtained through normalizing annealing—might offer a good alternative for forged parts undergoing machining and subsequent heat treatment of peripheral sections. With growing energy costs, a compromise in machinability may be balanced by a reduction in energy consumption resulting from the elimination of reheating. This study investigates industrially based processing strategies, including hot forging and continuous or interrupted controlled cooling, directly after controlled hot forging. Thus, the utilitarian goal was to determine whether it is possible to replace the traditional energy-consuming process of normalizing annealing by direct cooling.

**Figure 1:**
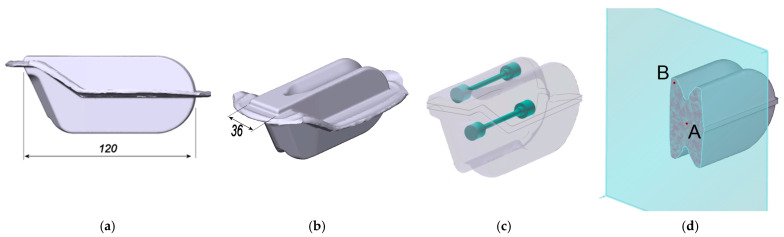
Geometry and major dimensions of the forged and heat-treated (HT) sample: (**a**) front view, (**b**) side view, (**c**) location of specimens, and (**d**) location of the cross-section used for the metallographic specimens and locations of the tracking points and tensile testing samples; A—the center (the lowest cooling rate), B—the corner (the highest cooling rate).

To ensure the similarity of process conditions, considering all factors affecting an industrial process, the research was conducted in situ on a real hot forging line, using an actual production part as the sample to be forged and tested. For this, the exemplary part, a tarmac cutter, made of medium-carbon microalloyed steel, was used. The basis for determining and selecting subsequent cooling conditions was a typical forging sequence designed for this part. The geometry of the sample part and its dimensions are shown in Figure 1, with the location of the specimens for tensile testing.

Unlike the majority of related studies, which investigate material–condition–property relationships through dilatometric thermal analyses [23,24], where results pertain to a specific location, this study aims to analyze cause-and-effect relationships in a full-scale part. Thus, various factors relevant to designing heat treatment strategies for different material–geometry combinations are taken into account. Hence, there are three levels of investigation involved in the study: (1) local identification of transformation products under specific cooling conditions, (2) estimation of cooling regimes in selected areas of a complex, full-scale part, and (3) full-scale physical modeling of selected cooling strategies, considering interactions such as deformation heat [25,26] or latent heat of transformation [27].

The first aspect involved designing the desired patch of cooling curves, and utilizing a numerical transformation hybrid model computed using the TTSteel code to select the proper start and interrupted cooling temperatures. The hybrid transformation diagram, which encompasses features of CCT and TTT diagrams, was found to be suitable for simulating continuous cooling with isothermal holds. The next part of the study involved experimental cooling tests of a full-size component. These tests were conducted in a continuous cycle in a special continuous cooling laboratory simulator replicating industrial conditions and employing assumed cooling strategies. Based on the modeling results, three regimes of direct cooling (DC) were realized within experimental tests for the same sample geometry:

1. Direct Cooling: Natural cooling immediately after hot forging without reheating (DC1),
2. Direct Cooling (modified strategy): Forging at the same temperature, followed by direct cooling with a revised strategy involving accelerated air cooling interrupted to produce a pseudo-isothermal plateau within the pearlite zone (DC2),
3. Direct Cooling using the same strategy for the modified forging regime, that is, a lowered forging temperature (DC3).

**Figure 2:**
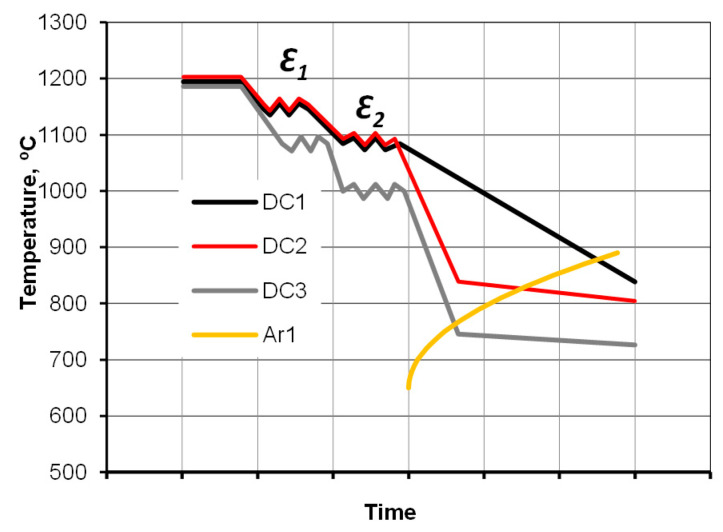
Graphical presentation of the research plan, where DC1 ÷ DC3 are direct cooling variants, ε_1_—forging operations, and ε_2_—trimming and sizing.

A schematic illustration of the experimental plan is shown in Figure 2. In addition to the direct cooling experiments, conventional normalizing annealing (NA)—a process involving reheating and natural cooling of as-forged samples—was carried out as a reference.

The DC1 variant utilized natural cooling in the air to cool the hot-forged samples. However, to provide slow cooling, it was conducted inside the cooling chamber housing the conveyor for transferring the forged parts. There, to reproduce the industrial process, the part being cooled was interacting with neighboring equally hot parts. The cooling scheme DC2 assumed that immediately after forging, the forging was cooled with vent-accelerated air below the stability temperature of austenite and then transferred to the same environment as DC1. As a result, an isothermal hold was obtained. Temperature equalization was meant to produce an increase in the temperature of the supercooled near-surface zone. The recalescence effect of the transformation brings about the temperature rise, resulting in a pseudo-isothermal patch of cooling in the pearlite zone. The properties of the resulting structure will depend on the degree of undercooling during the subsequent transformation and the nature of the temperature fluctuation, especially the duration of the pseudo-isothermal hold for diffusion-based processes. Thus, besides synchronizing the stoppage of accelerated cooling with transformation, one of the challenges is the amount of plastic work required to control the uniformity of the grain structure in the bulk. It also provides the necessary cooling rate for the transformation to initiate in the lower range of the pearlite zone. Since controlled cooling takes place immediately after hot deformation (with an interval for trimming), the as-forged microstructure is retained during cooling, thereby controlling the size of the pearlite colonies. Decreasing the temperature of the transformation for pearlite allows for low interlamellar spacing. The subsequent slow cooling favors the effects of carbon partitioning [28]. Although its impact depends on the weight of the part being forged, it contributes to the improvement in toughness and plasticity [29,30]. The austenite grain size will primarily result from microstructure evolution during dynamic recrystallization processes. In the vanadium-alloyed steel (used in the study), the additional influence of carbide precipitates on the kinetics of ferrite nucleation and growth must also be noted. As related studies [31] demonstrate, an extended holding time promotes the nucleation and growth of VC particles, thereby affecting their effectiveness in controlling microstructure.

For forging steels intended for heat treatment (quenching and tempering), the hot forging temperature plays a secondary role. Due to the high forming resistance and the presence of hard boron nitride particles, which are stable at elevated temperatures and detrimental to plasticity, forging is usually carried out at sufficiently high temperatures [32,33]. In the analyzed process, the forging stock is typically heated to a temperature of 1220 °C to reduce forging loads. Hence, for these tests, the forging temperature is lowered to 1190 °C. It allows the maximum temperature to be reduced to a range that ensures the formation of fine lamellar pearlite and maximum nucleation intensity of vanadium carbides [34,35]. The pearlite formed at relatively high undercooling is characterized by a low interlamellar spacing, thin lamella thickness, and small pearlite colony size. In addition, the cementite lamellae formed under such subcritical conditions may exhibit a higher density of defects, due to strain accumulation during phase transformation and reduced diffusion at low temperatures [18].

### 2.2. Physical Modeling

A typical controlled cooling line consists of several cooling fans. When the forging process is completed, the forged piece is transferred through the line where forced-air cooling occurs. The capacity of the fans results in air flow velocity, which produces the required cooling rate in cross-sections of the forged part. The number of fans (cooling zones) can vary depending on the size and weight of the forged part, as well as the required cooling rates.

**Figure 3:**
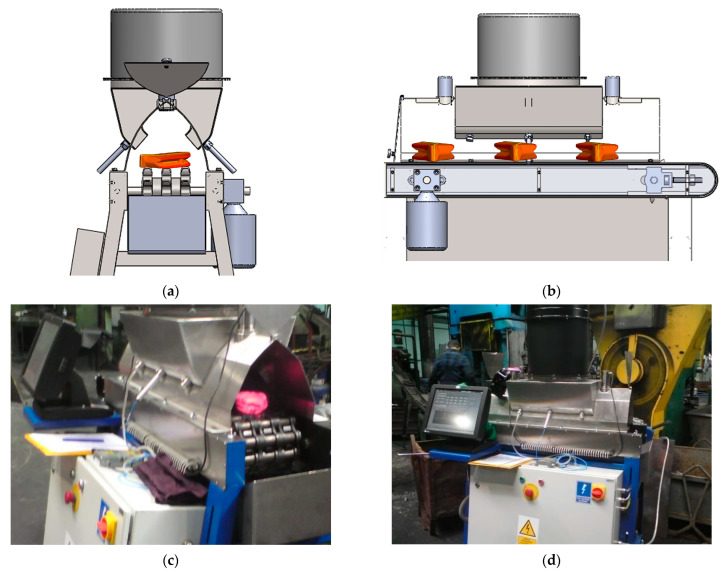
Laboratory cooling equipment QuenchTube. Model: axial view (**a**), side view (**b**), and experimental stand during industry in situ tests: axial view (**c**), and side view (**d**).

**Figure 4:**
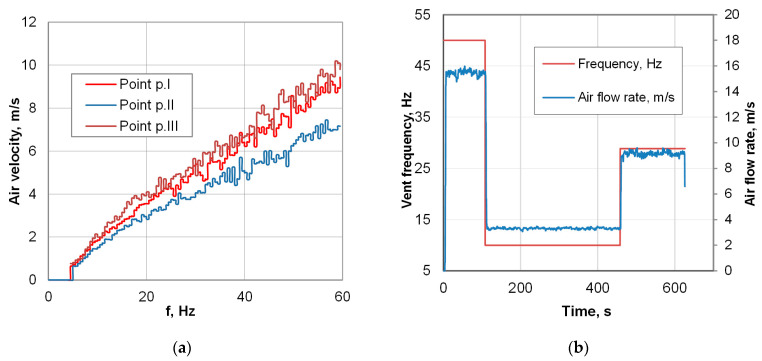
Air flow control in the cooling zone: (**a**) dependence of air rate on frequency, and (**b**) air-rate control schedule of the interrupted cooling cycle used in the study.

**Figure 5:**
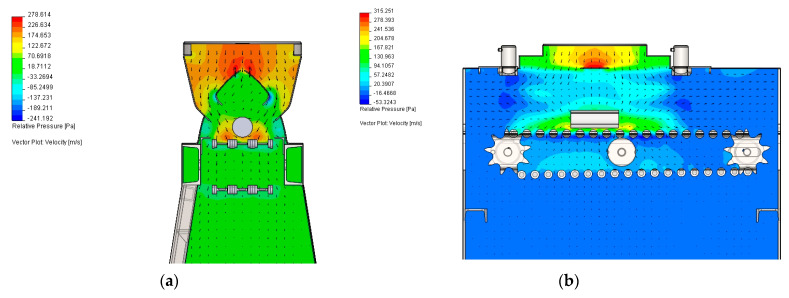
Pressure and velocity vectors (the arrows) in the cooling conveyor chamber: (**a**) front view, and (**b**) side view.

**Figure 6:**
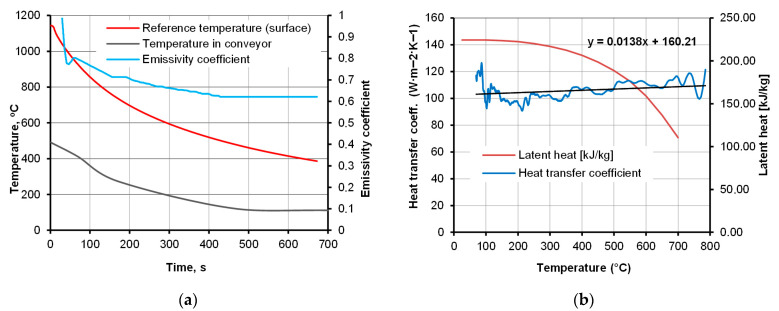
Dependence of selected boundary conditions used in FEM simulation of cooling on process parameters: (**a**) emissivity coefficient and heat transfer coefficient vs. temperature [36], and (**b**) environment temperature in the cooling chamber with consideration of neighboring samples.

The presented study utilizes special cooling equipment that simulates the action of a forced-air cooling line, the Quench Tube^TM^ (Figure 3). Several cooling zones, commonly found in industrial lines, are replaced with a single cooling zone featuring reversible conveyor movement. The actual temperature of the workpiece was measured by means of pyrometers, installed at both ends of the zone. The experimental line features only one cooling section, operated by a single vent; however, reciprocal movement enables the simulation of multi-zone cooling. At both ends of the line, laser pyrometers are mounted, which measure the instant temperature between consecutive passes. From a technical standpoint, the realization of the experimental part necessitates elaborating on the process conditions, such as the environmental temperature in the cooling chamber and vent frequency. The assumed cooling rate and thereby required time–temperature regime was realized with controlled conveyor belt velocity and fan frequency (Figure 4) to provide the necessary pressure and air velocity, whose distribution in the cooling chamber is presented in Figure 5. After deformation, the heat-treated part enters the cooling line, with its temperature measured. As the direct cooling of forgings is associated with fast temperature drops, reliable temperature measurement requires the emissivity coefficient’s temperature function. The determination of the dependence of emissivity on the temperature of the forged part was achieved by automating a classic fall-behind feedback control system [36]. The time dependence of emissivity presented in Figure 6a was used in FEM simulations and temperature remote measurements.

### 2.3. Numerical Modeling

Contrary to the conventional heat treatment, direct cooling does not involve an austenitizing temperature. Once the workpiece is heated up, the temperature is controlled by deformation conditions and subsequent cooling. The forging-end temperature is also the start of the cooling process, and it is heating that allows the temperature to be corrected. Due to difficulties in acquiring data from measurements, the knowledge of temperature required for designing the cooling strategy depends on modeling.

FEM was conducted using the QForm3D commercial code, assuming a rigid-viscoplastic model of a deformed body. The simulation involved thermo-mechanical analysis with the use of the Voronoi cells method for approximation of the calculated fields of thermal and mechanical parameters. A tetrahedral element mesh with adaptive remeshing was used. A refined mesh defined by an adaptation factor of 2 was used in the domain with higher gradients of the solution. Frictional conditions were described by Levanov’s friction model, with a friction factor of 0.4. The remaining boundary conditions were based on industrial process conditions, including a hammer energy of 26 kJ and a tool temperature of 180 °C.

The equations describing the heat transfer model can be found in [37,38,39]. Heat transfer boundary conditions were derived from a series of additional calculations within related studies [21,36,40]. The air velocity in the cooling chamber (Figure 5), used in the simulation of the modified cooling strategies DC2 and DC3, was obtained from a related study [40]. The heat transfer coefficient and latent heat values of the austenite transformation (Figure 6b) are derived from related work [39]. Considering that in the real process, consecutive forged parts are being fed into the conveyor, the effect of their mutual interaction was also taken into account. With the assumption of the average separation of forgings, the environment temperature at 15 cm from the surface was calculated based on the Stefan–Boltzmann law. Plot of its function at the time of cooling is shown in Figure 6a.

### 2.4. Material Considerations

The material used in the study was a medium-carbon steel, 35CMnTiB4 (Table 1), with boron and microalloying elements, including Ti, V, and Nb. The composition of the steel, presented in Table 1, is a modification of the commonly used medium-carbon grade 36MnB with great potential for grain refinement and precipitation hardening by the action of microalloy additives. Its effectiveness is controlled by the amount of strain in the multistage hot forging process, forge-end temperature, and controlled cooling.

The strategy assumed includes soaking high enough to dissolve possible boron precipitates and accommodating time–temperature conditions suitable for strain-induced precipitation. From the standpoint of grain size control as well as the detrimental effect of unwanted coarse precipitates, relative proportions between the contents of B, N, Al, and Ti are of primary significance. Starting from stoichiometric ratios, simple rules must be followed in the prediction of the presence of boron, vanadium, and titanium nitrides (carbonitrides):

(1) Minimum Ti content can amount to %Timin ≈ 3.4%N. Ti forms very stable TiN nitrides with a high solvus temperature. Thus, if all available N will be consumed by TiN formation, B will remain in the solid solution, inhibiting the formation of ferrite and pearlite at the austenite grain boundaries by blocking nucleation sites.
(2) Considering the presence of Al and V, the minimum amount of Al to bind N is 1.93%Al, ≈1.93%N. Formation of AlN (similarly to TiN) promotes B activity on grain boundaries, enhancing hardenability [41]. On the other hand, excessive concentration of coarse aluminum or titanium nitrides may deteriorate impact strength and machinability.

In the primary structure, TiN precipitates are generally formed in the liquid melt or during solidification before break-up of the cast structure. NbCN, NbN, NbC, and TiC precipitates form in the austenite regime. VC precipitates are formed in austenite–ferrite or ferrite range during subsequent cooling [42], pinning the grain boundaries and controlling grain growth of transformed austenite [43]. It can be concluded from the above observations that a preheating temperature of 1200 °C ensures that almost all of these precipitates—except TiN, which has a solvus point as high as 1500 °C [44,45]—are dissolved and available for precipitation hardening upon cooling.

## 3. Results and Discussion

### 3.1. Characterization the Workpiece Material

**Figure 7:**
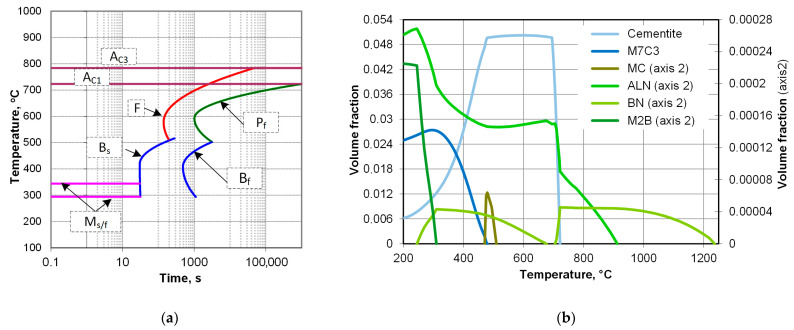
Numerical estimation of transformation products for 35CMnTiB4 steel used in the study: (**a**) TTT diagram, and (**b**) stability of equilibrium phases calculated in Thermocalc.

**Figure 8:**
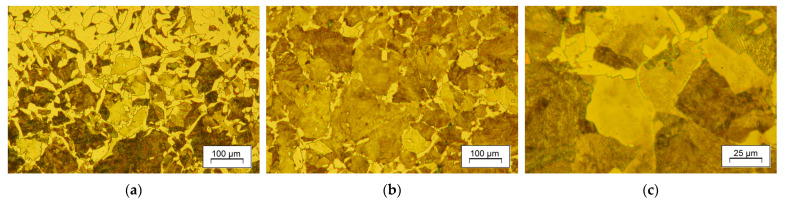
Microstructure of forging billet in the as-received condition: (**a**) surface, (**b**,**c**) the axis.

As an aid for designing the cooling curve paths, the TTT diagram, calculated with the TTSteel v.2.1 software (ITA s.r.o.), is shown in Figure 7a. There can be several significant types of precipitates present in the alloy considered, as indicated by the equilibrium diagram calculated in Thermocalc (Figure 7b). The microstructure of the material in as-received condition is shown in Figure 8. It consists of a ferritic–pearlitic structure, which exhibits good uniformity and a grain size of about eight to nine ASTM, with ferrite forming mainly as grain-boundary (GB) ferrite. However, due to decarburization in the subsurface regions, ferrite tends to prevail and exhibits an acicular morphology. In general, if the condition of the workpiece is to be rated, it is favorable for high-strain-rate forgeability and for achieving uniform properties after forging and controlled cooling.

### 3.2. Analysis of Thermo-Mechanical Factors

Unlike traditional heat treatments, in which naturally cooled forged parts are reheated and held for austenitizing, the forge-end temperature is equivalent to the austenitizing temperature. As such, it is one of the degrees of freedom to be considered to control the cooling. However, as it results from the forging regime, its minimum and maximum values, as well as its gradients in the bulk, can only be controlled through the proper design of the entire forging cycle. To determine the starting point for cooling, including heat generated by plastic deformation, finite element modeling (FEM) was used.

#### 3.2.1. FEM of Hot Forging and Direct Cooling

One of the relevant parameters to consider was the amount of plastic work, which is an indirect indicator of grain refinement and homogeneity, represented by the effective strain. Information of significance is also the division of the plastic deformation between forging stages. In the event of a detrimental effect due to an inadequate driving force for producing fine grain or excessive heat generation, revising the forging stages could be suggested.

**Figure 9:**
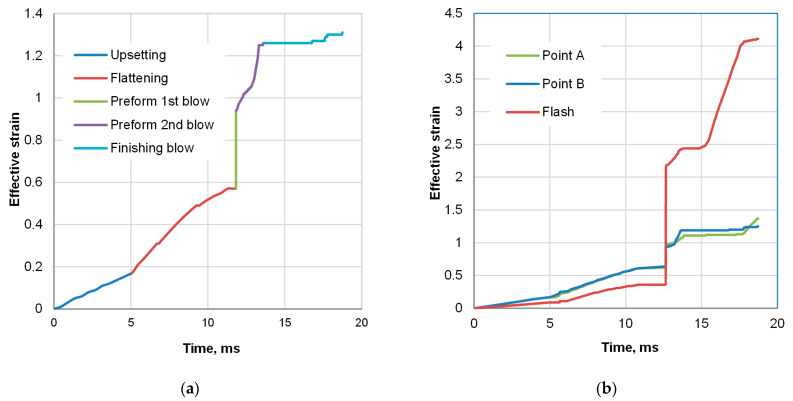
FEM-calculated evolution of effective strain: (**a**) contribution of consecutive forging stages, (**b**) comparison of effective strain in the center of a sample (point A), the ribs (point B), and in the flash.

**Figure 10:**
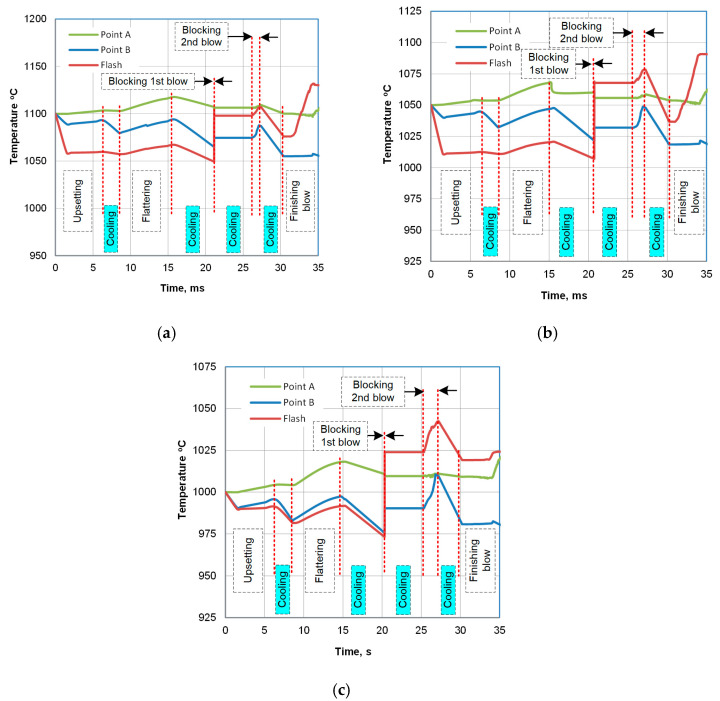
FEM-calculated evolution of temperature in consecutive forging stages, indicated by red dashed lines, in point A, and comparative analysis of temperature changes in the center of a sample (point A), the ribs (point B), and in the flash for initial temperature 1100 °C (**a**), 1050 °C (**b**), and 1000 °C (**c**).

To observe the evolution of both strain and temperature, tracking points A and B were defined in the mesh (Figure 1c). The evolution of strain at a point is shown in Figure 9a, with indication of consecutive hammer strokes. Figure 9b illustrates its development in the selected locations. Despite different instant changes, the final level of effective strain in the observed locations is comparable. The results of the temperature analysis are shown in Figure 10. According to the modeling results, the deformation heat raises the temperature above 1190 °C, and the forge end is slightly above 1120 °C. After the subsequent trimming stage, the temperature falls to about 1105 °C, at which point cooling begins.

Another noteworthy piece of information derived from the simulation is that plots of temperature evolution during the entire forging cycle differ. In point A—in the interior of the bulky sample—it does not fall much below the start-forge point. For instance, at 1100 °C, the temperature is kept above 1090 °C. Contrary to the center, in the ribs (point B), the temperature is about 30 degrees lower due to the die chill, despite the rise caused by high-strain-rate deformation heat generation. In addition to geometry reasons, the fact that die-chill effect in the center of the part is more negligible is as a result of the heat flux towards the center from the flash, which is the hottest region in the volume of the sample part (point C in Figure 9b and Figure 10). Hence, the two selected locations, A and B, came up as two extreme points for observation: point A (interior) has the highest starting temperature with the lowest cooling rate and point B (the ribs) has the location of the lowest starting temperature with the predicted highest cooling rate.

For consideration of the modified forging regime, initial temperatures of 1050 °C and 1000 °C were also assumed. While 1050 °C is a case of choice, the remaining ones end close to the non-recrystallization point, which according to the Boratto equation [42,43], exceeds 1000 °C. A non-recrystallized structure causes a shift in the transformation start temperature [35,46,47], shortening the time by an order of magnitude [48] with a risk of forming upper bainite.

**Figure 11:**
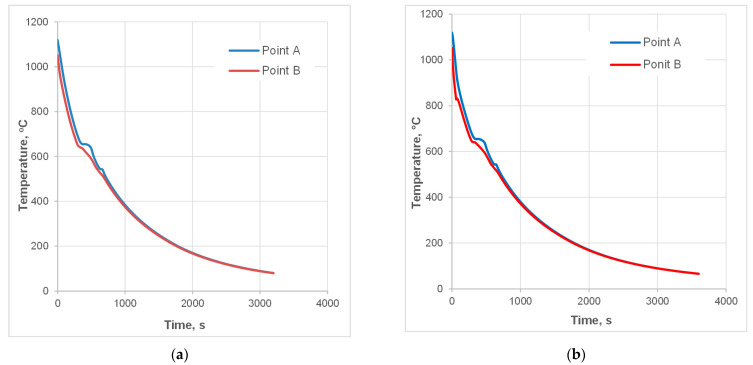

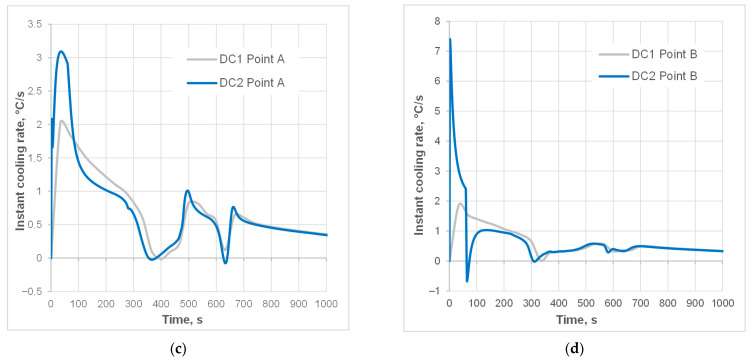
FEM-calculated temperature during direct cooling: (**a**) continuous cooling (DC1), (**b**) continuous interrupted cooling (DC2), (**c**) instantaneous cooling rate in the center of the part, and (**d**) instantaneous cooling rate in the subface of the ribs.

As a continuation of the hot forging process, the cooling phase was simulated, taking into account a short interval to represent the trimming operation. The analysis of two cooling strategies, DC1 and DC2, is shown in Figure 11a and Figure 11b, respectively. The simulation results indicate that noteworthy differences in the temperature plots occur primarily in the undersurface area, with little or no effect on the temperature in the center of the part. However, the time derivative of temperature reveals a difference in cooling rates at the two points (Figure 11c,d). The application of accelerated air increased the cooling rate at point B (undersurface) by a factor of two (Figure 11d). At the same time, this change in strategy had no noticeable effect on the cooling rate at point A (the core), which indicates that the cooling rate remained essentially unchanged throughout the bulk of the part, which opens up promising possibilities for selectively controlling surface and subsurface hardness, while maintaining a softer core. A recapitulation of the estimated process parameters, along with the process conditions resulting from industrial limitations, is presented in Table 2.

#### 3.2.2. Controlled Cooling Experiments

**Figure 12:**
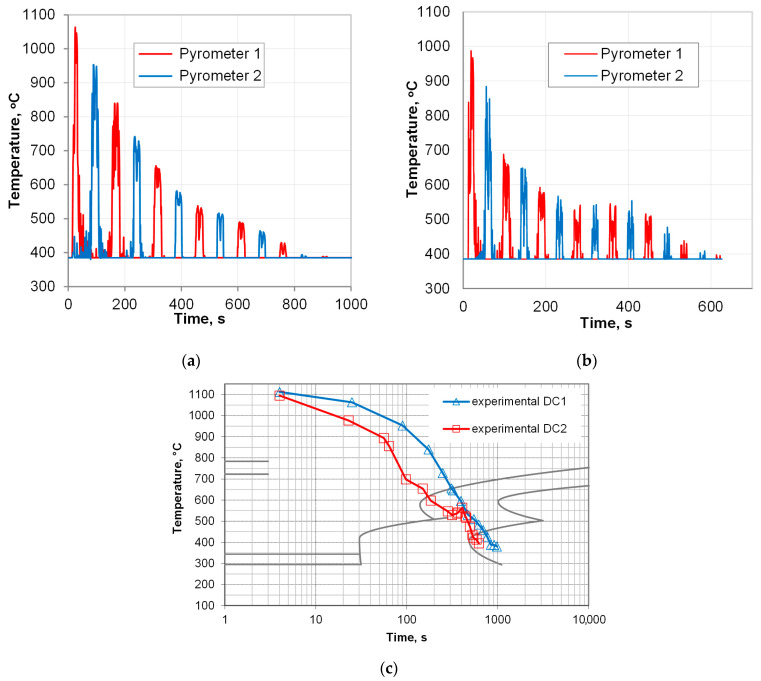
Temperature pyrometer measurement records from in situ experimental tests: (**a**) natural continuous cooling, and (**b**) interrupted accelerated air cooling, (**c**) pyrometer readings during the experimental cooling tests DC1 and DC2 superimposed on the TTT diagram.

Based on the simulation results, experimental tests were conducted. Due to limited possibilities of thermocouple measurements, pyrometers were used, and only surface records were available. The tips of the pyrometers’ indications represent the temperature on the surface of the samples while passing the pyrometer gauge spots P1 and P2 (Figure 12). The temperature plot shows the course of direct cooling starting from 1090 °C for cooling with slow air in an isolated cooling chamber (Figure 12a) and for interrupted fan cooling (accelerated air with a velocity of 15 m/s with subsequent 180 s interval for temperature equalization in the subface zones), as shown in Figure 12b. For more straightforward elucidation of the temperature change during the two cooling experiments, a plot created by joining the tips of the pyrometer indications is presented in Figure 12c. Plotting these curves on a logarithmic scale better highlights the differences resulting from accelerated cooling. In particular, a significant difference in the temperature of the onset of the pearlitic transformation can be observed. Since the interlamellar spacing decreases as the reaction temperature reduces (and as the imposed velocity is increases—see Figure 11c,d), a difference of over 60 °C is bound to slow down redistribution of carbon and the substitutional elements between the product phases at the reaction front [28,49]. Thus, according to the Zener–Hillert law, a smaller spacing in pearlite was expected, which was confirmed by later microstructure characterization. The pseudo-isothermal hold caused by interrupted cooling, as the temperature on the surface dropped to 600–550 °C, brought about a slight temperature rise. This provided the appropriate conditions for the growth of the lamella, preventing the formation of upper bainite, as illustrated in Figure 12b.

#### 3.2.3. Evaluation of the Results

**Figure 13:**
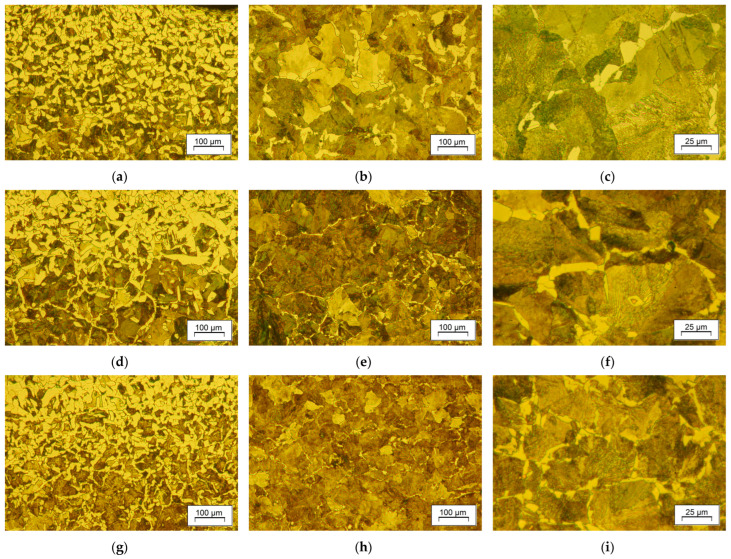
Microstructure of the samples control-cooled directly from the hot-forging temperature for different cooling strategies: (**a**–**c**) DC1, (**d**–**f**) DC2 and (**g**–**i**) DC3, where (**a**,**d**,**g**) undersurface area, and (**b**,**c**,**e**,**f**,**h**,**i**) central region.

**Figure 14:**
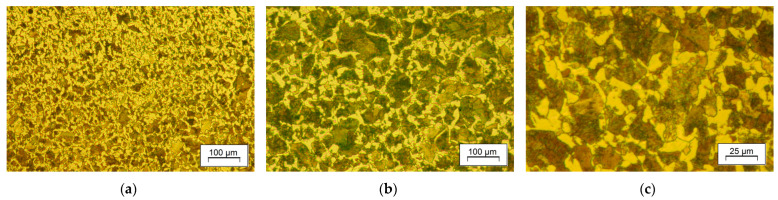
Microstructure of conventionally normalized sample: (**a**) undersurface area, and (**b**,**c**) central region.

The effect of forging and direct cooling is reflected in the microstructure of the forgings after varying cooling variants. In Figure 13, microstructures representative of each applied cooling strategy, direct-cooled (Figure 13a–i) and conventionally normalized (Figure 14) materials, are compared. The microstructure of all analyzed samples composed of ferrite and pearlite (F-P), with differences in the ferrite form and visually undetectable variations in pearlite morphology. One of the common things about each series is decarburization in the surface areas (Figure 13a,d,g), which is a natural inclination of this grade during forging at increased temperatures to enable forging with soft blows of the hammer. In the results, the peripheral zones of the part exhibited a higher ferrite content due to natural cooling after standard hot forging. These areas are dominated by polygonal ferrite. In the representative region of the sample, grain-boundary ferrite prevails.

Application of accelerated cooling rate at the entry of the cooling chamber resulted in higher content of pearlite with thinner lamellae of the grain-boundary ferrite (Figure 13e,f,h,i). Pearlite is more refined and has a darker shade in white-field micrographs, which may result from a smaller interlamellar spacing or incomplete restoration of grains during dynamic recrystallization. Pearlite colonies featuring material processed with the DC3 cooling strategy (Figure 13h,i) are clearly finer, which is an anticipated result of decreasing forging range and the start of the pearlitic transformation. As indicated by estimation with the linear intercept method, the grain size of the material after controlled processing DC3 is not much bigger than that of the conventionally normalized steel, as presented in Figure 14. Multiple transformations during heating and cooling of the reference sample (NA) resulted in grain refinement and uniformity, which is superior to that of the direct-cooled samples. It achieved the smallest value of the linear intercept, reaching less than 20 μm, which is slightly less than the smallest among direct-cooled structures. What attracts attention is the relative similarity of the samples’ microstructure. It tends to show that random variation in process conditions has a limited effect on microstructure development under slow cooling with a relatively high start of austenite transformation. That prevents unintended deterioration of properties due to grain growth or upper bainite hazard, offering a considerable enhancement of strength.

**Figure 15:**
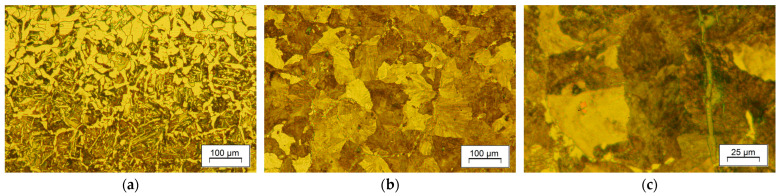
Microstructure of forged steel at stage of flattening: (**a**) undersurface area, and (**b**,**c**) central region.

The ultimate estimator of the obtained microstructure is the mechanical properties. It is hardly possible to equally satisfy competing material considerations through controlled cooling. Dissolution of precipitates with no grain growth, decarburization, or shape progression, while simultaneously controlling strain uniformity and temperature fields, is challenging. It affects material response. For instance, for a favorable metal flow that provides proper fill of the die impression, the preforming stages are determined with a narrow margin of variation in deformation degree. That imposes maximum strain in upsetting and flattening. In the aftermath, relatively coarse-grained pearlite forms (Figure 15), which can be hard to refine during subsequent forging stages, especially when the non-recrystallization temperature is relatively high. Substantial grain refinement was achieved through the transformation of overcooled austenite. The transformed structure of F-P exhibits a smaller grain size, which was confirmed by the stereographically estimated linear intercept values (Table 3). The modification of the forging and direct-cooling regime also had an impact on the morphology of pearlite and is associated with the mechanical behavior of the material. The metallographic estimation of spacing in pearlite, using the Vander Voort method [50,51] in combination with observed grain refinement, justifies the observed strength enhancement produced by DC2 and DC3 in relation to the as-forged condition or DC1 strategy (slow cooling in the tube). Despite the evident scatter of the obtained values, attributed to the random orientation of pearlite grains in relation to the polish, the spacing values are smaller for the higher cooling rates of DC2 and DC3 compared to natural cooling. The significant effect of grain size (colony) and interlamellar spacing in pearlite confirms their positive influence on strength and plasticity. However, the multi-aspect interaction of concurrent factors does not allow for plain rationalization of the contribution of a single factor to the final properties. For example, the fine-grained microstructure of conventionally normalized steel exhibits slightly lower strength properties in relation to the direct-cooled samples. It suggests the significance of ferrite morphology on tensile properties. As shown in Figure 13, a lower temperature of austenite decomposition also resulted in thinner lamellae of grain-boundary ferrite.

**Figure 16:**
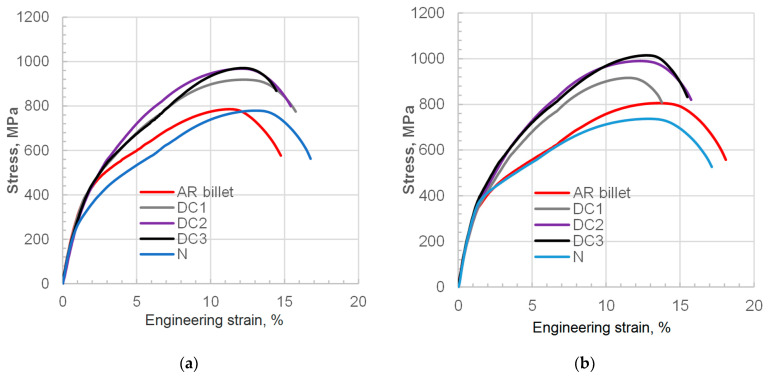
Mechanical testing results obtained for variable cooling conditions in locations under consideration: (**a**) point A, and (**b**) point B.

The results of mechanical testing on specimens derived from cooled forgings are presented in Figure 16, grouped by location (points A and B) and summarized in Table 3. As expected, neither the microstructure nor the tensile properties can be considered literally comparable to the annealed condition. Nevertheless, the treatment strategies presented can still be satisfactory in terms of machinability and the overall combination of properties required from 35MnB4 for post-forging machining and further heat treatment. Several factors should be considered in this light. Firstly, this steel grade is not easy to machine in general. Relatively high carbon produces a significant amount of hard phases, and the microadditives contribute to precipitation hardening by controlling the dispersion of carbonitrides and austenite grain as a result. That makes it suitable for thicker-section applications, where wear of machining tools is less important, e.g., machining of near-net forged parts. Considering the moderate machinability of this steel grade, as well as the limitations in softening it for machining, the observed increase in hardness may be an acceptable compromise in terms of the energy savings gained from eliminating reheating. Moreover, as indicated in micrographs derived from surface and undersurface areas, this grade is prone to decarburization when soaked at high enough temperatures for the precipitates to be dissolved. Therefore, for higher accuracy class die forgings, the allowances cover the ferrite-rich decarburized layer, while the bulk may be provided with target strength. As indicated, even without subsequent quenching–tempering heat treatment, the tensile strength after controlled air cooling can reach or exceed 1000 MPa (in the case of DC3, 1015 MPa), which offers additional benefits. To sum up, in the era of high energy costs, gas, and emission markups, shortening the technological chain by removing energy-consuming thermal treatments may help to balance the inevitable shortcomings of hardness that is higher than that of conventionally normalized materials in the machining process.

## 4. Conclusions

The presented study was focused on developing post-forging processes for medium-carbon boron-alloyed steel and investigating their impact on microstructure and mechanical properties directly from the hot forging stage. A comparative analysis of conventionally normalized and direct-cooled medium-carbon microalloyed steel with boron addition was conducted to assess its potential for achieving tailored properties. The results obtained allow for the following conclusions:

The direct-cooled microstructure, consisting of grain-boundary ferrite and pearlite, exhibited tensile properties of Re ≈ 610 MPa, Rm ≈ 910 MPa, and A5 ≥ 12%. Although slightly lower ductility was observed compared to the normalized condition (Rm ≤ 780 MPa, Re ≤ 460 MPa, A ≥ 14%), machinability and strength were notably improved. The study highlights direct cooling as an energy-efficient alternative to conventional normalizing for producing high-duty 35MnTiB4 steel components. The strength properties of the forged samples subjected to direct cooling was 100–150 MPa higher than conventionally normalized ones, but with a reduction in elongation of only 2%, which favors machinability considerations with simultaneous cost savings benefits.

Microstructure parameters resulting from the assumed direct cooling strategies, such as interlamellar spacing, pearlite colony size, and the morphology of grain-boundary ferrite, indicate evident dependence on the applied thermo-mechanical processing conditions. Despite apparent similarity, the spacing between carbonate lamella indicated significant dependence on decreased forging temperature and application of accelerated cooling. Direct-cooled samples exhibit slightly higher pearlite content with finer colonies than naturally cooled ones, where ferrite is more dominant, especially near decarburized surface areas. In comparison, conventionally normalized steel exhibits a more refined and uniform grain structure, but it shows similar or thicker interlamellar spacing.

The technical realization of controlled cooling directly after hot forging in the mass production of hot-forged parts requires us to address several technological aspects. As the microstructure condition and temperature fields are inherited from the forging process, the forging regime and the cooling conditions must be determined in terms of prior deformation. One of the challenges in this case was synchronizing the cooling stages with the onset of the Feγ→Feα transformation to take advantage of the generated latent heat, in order to achieve temperature equalization during the isothermal hold, thereby producing the required morphology of ferrite, interlamellar spacing, and size of pearlite colonies.

The presented results indicate technological considerations for employing direct cooling in hot-forged steel 35CMnTiB4 with microalloying elements to achieve properties comparable to those of normalized steel through direct cooling of hot-forged parts.

## Figures and Tables

**Table 1:** Chemical composition of the steel 35CMnTiB4 used in the study.

%C	%Cr	%Si	%Mn	%Mo	%Ni	%Ti	%V	%B	%Nb	%Sn	%N_2_	%Al	%P	%S	Fe
0.35	0.37	0.27	1.36	0.007	0.06	0.034	0.005	0.002	0.05	0.013	0.0105	0.02	0.013	0.011	bal.

**Table 2:** Selected process conditions of the experimental tests.

Treatment Strategy	Designation	Cooling Strategy	Forging Temperature	Effective Strain		Cooling Start, °C		Max. Cooling Rate, °C/s	
			Tε_1_	A	B	A	B	A	B
**conventional** **normalizing**	NA	Reheating + air	1200 °C	1.45	1.41	880 °C	870 °C	1.8	3.2
**Direct cooling 1**	DC1	Air (tube)	1150 °C	1.45	1.41	1105 °C	1055 °C	2.1	3.2
**Direct cooling 2**	DC2	Acc air + hold	1150 °C	1.45	1.41	1105 °C	1055 °C	4.1	7.4
**Direct cooling 3**	DC3	Acc air + hold	1090 °C	1.45	1.41	1060 °C	1020 °C	3.5	7.0

**Table 3:** Mechanical properties of the forged samples made of modified 35MnB4 steel subjected to different controlled cooling strategies directly after forging.

Sample	Specimen	Rm, MPa	Re, MPa	A_10_, %	Interlamellar Spacing, μm	Grain Size, ^(2)^ μm
As-received ^(1)^	A	786.5	483.1	13.4	264 ± 63	26.82 ± 3.2
	B	806.0	475.1	16.2		
conventional normalizing (NA)	A	779.6	424.6	14.6	208 ± 38	18.65 ± 1.7
	B	736.8	458.4	15.8		
Direct cooling 1 (DC1)	A	919.2	606.2	13.7	245 ± 22	36.28 ± 3.5
	B	916.3	647.2	11.9		
Direct cooling 2 (DC2)	A	968.1	656.2	13.4	222 ± 39	26.32 ± 3.5
	B	989.4	648.0	13.2		
Direct cooling 3 (DC3)	A	971.0	605.8	12.2	190 ± 48	19.18 ± 2.8
	B	1015.0	652.5	12.5		

Designations of locations for ^(1)^ as-received billet: A—core, and B—surface. ^(2)^ Average linear intercept (in μm).

## Data Availability

The original contributions presented in this study are included in the article. Further inquiries can be directed to the corresponding author.
